# Impact of Two Flap Advancement Techniques and Periosteal Suturing on Graft Displacement During Guided Bone Regeneration

**DOI:** 10.1111/cid.13434

**Published:** 2025-01-15

**Authors:** Clemens Raabe, Emilio A. Cafferata, Emilio Couso‐Queiruga, Vivianne Chappuis, Ausra Ramanauskaite, Frank Schwarz

**Affiliations:** ^1^ Department of Oral Surgery and Implantology Goethe University Frankfurt am Main Germany; ^2^ Department of Oral Surgery and Stomatology, School of Dental Medicine University of Bern Bern Switzerland; ^3^ Oral Peri‐Implant Research Group, School of Dentistry Universidad Científica del Sur Lima Peru

**Keywords:** alveolar ridge augmentation, bone substitutes, cone‐beam computed tomography, dental implants, grafts, guided bone regeneration, surgical flaps

## Abstract

**Objectives:**

This preclinical ex vivo porcine study aimed to evaluate the effects of two flap advancement techniques and periosteal suturing (PS) on graft material displacement during primary wound closure in guided bone regeneration (GBR). Secondary objectives included assessing flap advancement and the impact of soft tissue characteristics on graft displacement.

**Materials and Methods:**

Standardized two‐walled horizontal bone defects were created in second premolar sites of pig hemimandibles. Sites were randomized to using either full‐thickness flaps with modified periosteal releasing incisions (MPRI) or combination flaps using the mucosal detachment technique (MDT), both with and without PS. Cone‐beam computed tomography was used to measure changes in graft material thickness (GMT) at seven incremental levels (L0–L6) relative to the implant platform, before and after primary wound closure. Keratinized mucosa width (KMW), flap thickness (FT), and flap advancement (FA) were also recorded.

**Results:**

Sixty‐eight horizontal bone augmentation procedures were performed on 34 pig hemimandibles, divided into four groups (MDT+PS, MDT‐PS, MPRI+PS, MPRI‐PS). Mean overall change in GMT at L0 was −24.5% ± 14.0% for MPRI and − 23.0% ± 14.3% for MDT (*p* ≥ 0.085). PS reduced graft displacement (−14.2% ± 11.5%) compared with no PS (−33.2% ± 16.9%, *p* < 0.001). FA was 8.3 ± 1.1 mm (MPRI) and 8.3 ± 1.5 mm (MDT). The mean KMW was 6.8 ± 0.9 mm, and FT ranged from 0.8 to 1.6 mm.

**Conclusions:**

PS significantly reduced graft material displacement during primary wound closure, while flap advancement techniques and soft tissue characteristics had no impact on graft stability. Both surgical techniques provided sufficient flap advancement for primary wound closure.

## 1 Introduction

Tooth loss often results in local hard and soft tissue deficiencies because of wound healing and subsequent tissue remodeling, challenging tooth replacement therapy by dental implants [1, 2, 3]. To create an environment conducive to dental implant therapy, various techniques for tissue regeneration have been developed to reestablish sufficient hard and soft tissue volume at edentulous sites. In tissue regeneration, horizontal bone augmentation is most frequently performed using guided bone regeneration (GBR), with clinically validated long‐term efficacy and volumetric stability of GBR procedures following graft material integration [4]. These surgical techniques aiming at increasing tissue volumes, require tension‐free and primary wound closure to protect the grafted site from bacterial infection and provide stability, thereby ensuring successful outcomes [5].

To achieve primary wound closure, various flap advancement techniques have been proposed in the literature [6]. From these, full‐thickness flaps are frequently utilized, involving the blunt dissection of a mucoperiosteal flap from the bone, accompanied by one or more periosteal‐releasing incisions (PRI) with a depth of 1–3 mm, or 0.5 mm and lateral stretching (modified PRI [MPRI]) to facilitate adequate flap advancement [7, 8]. However, some authors prefer the use of combination flaps for certain clinical scenarios to achieve sufficient soft tissue advancement. These methods, such as the periosteal pouch flap or mucosal detachment technique (MDT), involve the elevation of a split‐thickness flap followed by local and pouch‐like deperiostation of the alveolar bone at the graft site [9, 10]. Compared with full‐thickness flaps, the resulting pouch is hypothesized to favor the containment and compartment of graft materials used for horizontal bone augmentation procedures, thus limiting micro‐movements and providing stability and more predictable healing [10]. A recent randomized clinical trial found the periosteal pouch flap to be as effective as the autogenous bone block technique in achieving bone gain during horizontal augmentation but with lower postoperative complication rates [11].

Most studies in bone regeneration focus on the effectiveness of volume gain and compare the immediate postoperative dimensions of the site to the follow‐up using 3D imaging [12]. However, this methodology fails to account for the potential intraoperative graft material displacement, which may result from tissue handling during primary wound closure, as indicated by recent studies [13, 14]. Indeed, particulate graft materials and resorbable membranes are particularly prone to collapse during flap repositioning, which results in a significantly reduced thickness of the graft material in the crestal area of the augmented site. To address this, investigations into different material combinations found that block grafts, L‐shaped collagenated xenografts, and membrane fixation using pins can significantly reduce graft displacement during wound closure [13, 14, 15]. However, the placement of pins or screws for membrane stabilization poses risks of injury to adjacent anatomical structures, such as tooth roots, the mandibular canal, or the maxillary sinus. Alternatively, periosteal suturing (PS) using absorbable suturing materials has been described to stabilize the bone graft while minimizing the need for second‐stage retrieval surgeries and reducing damage to adjacent vital anatomical structures during the insertion of pins or screws [16].

Nevertheless, the influence of the soft tissue compartment by the means of flap advancement technique and membrane stabilization using PS on the displacement of the graft materials during primary wound closure in GBR procedures is yet uninvestigated. Therefore, the primary aim of this ex vivo study was to evaluate the influence of two flap advancement techniques on graft material thickness (GMT) during primary wound closure in simultaneous GBR procedures. The secondary aims were to evaluate the effect of membrane stabilization by PS and the local phenotypical soft tissue characteristics on graft displacement. Finally, the null hypothesis stated that the flap advancement technique (H01), membrane stabilization using PS (H02), and the phenotypical characteristics of the soft tissues (H03) would have no effect on the displacement of the graft materials.

## 2 Materials and Methods

### 2.1 Ex Vivo Models and Study Groups

Ethics approval for this ex vivo study was not required. This investigation was conducted at the Department of Oral Surgery and Implantology, Goethe University, Carolinum, Frankfurt am Main, Germany from May to August 2024. Fresh pig hemi‐mandibles from 30‐week‐old specimens, sourced from a local butcher, were used to create a standardized defect at the second premolar site. These defects were augmented using GBR for simultaneous horizontal bone augmentation following random assignment to either one of two flap advancement techniques. Within each site, two GBR procedures were performed sequentially with or without membrane stabilization by PS, in a randomized order. This design resulted in four study groups, each with equal sample sizes:

– MDT without PS
– MDT with PS
– MPRI without PS
– MPRI with PS

Before surgery, site‐specific soft‐tissue phenotypic characteristics were recorded at the region of interest (second premolar site), including keratinized mucosa width (KMW), using a PCPUNC15‐probe and flap thickness (FT) by transmucosal horizontal probing using an endodontic file at 3, 6, and 9 mm (FT3, FT6, and FT9) apically from the gingival margin.

### 2.2 Flap Advancement and Bone Defect

**FIGURE 1:**
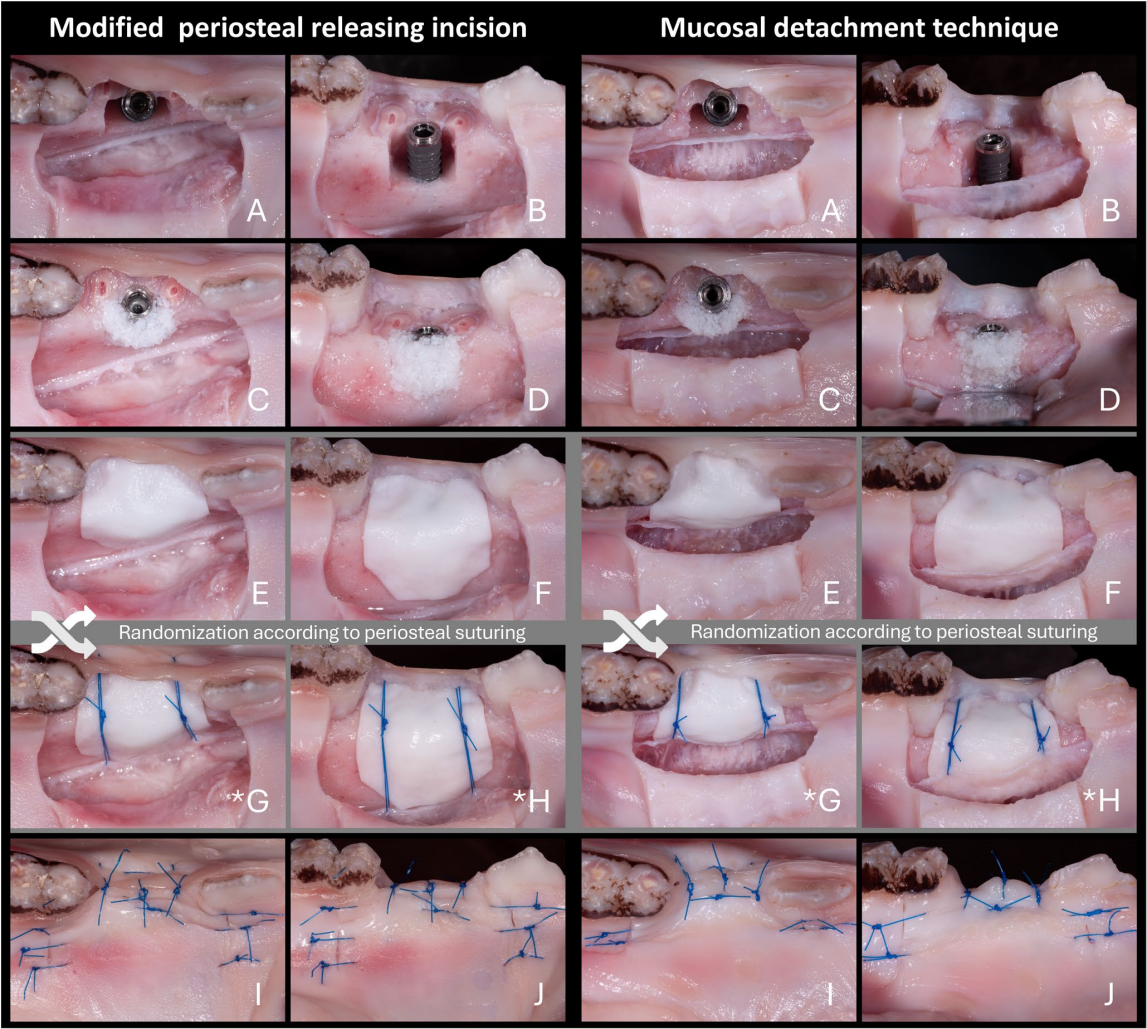
Flap advancement techniques using the modified periosteal releasing incision and mucosal detachment technique shown from occlusal and lateral views (A, B). Particulate deproteinized bovine bone mineral placement (C, D). Coverage with a native collagen membrane, without membrane stabilization (E, F), and with PS (G, H). For visualization purposes, PS is displayed using a colored, non‐absorbable suture (*). Primary wound closure (I, J).

All surgeries were performed by a single board‐certified and experienced oral surgeon (C.R.), including the placement of particulate bone substitutes, collagen membranes, and periosteal sutures (when required). The surgeries were initiated with one of the following flap advancement techniques (Figure 1):

1. MPRI: A trapezoidal flap design involving a sulcular incision at the second premolar, extended to the adjacent teeth with two slightly divergent and 10 mm long vertical releasing incisions, was performed. A full‐thickness flap was bluntly detached from the bone to the base of the flap using a periosteal elevator. Subsequently, a perpendicular periosteal incision with a depth of 0.5 mm was made horizontally at the base of the flap. The buccal flap was advanced by bluntly stretching the tissues along the horizontal incision line with the back end of the blade in a sweeping motion [7].
2. MDT: A trapezoidal flap design involving a sulcular incision at the second premolar, extended to the adjacent teeth with two slightly divergent vertical releasing incisions up to 1 mm apically to the mucogingival junction (MGJ), was performed. A full‐thickness flap was raised, slightly passing the MGJ. At the level of the MGJ, a periosteal incision with a depth of 0.5 mm was made. From this incision line in an apical direction, the submucosa was bluntly detached from the periosteum in an apical direction using a semi‐dull instrument. Subsequently, the periosteum was detached from the underlying bone using a periosteal elevator, creating a small buccal pouch that corresponded to the bone defect to be prepared (L. [9, 17]).

After completing the flap design and advancement, a coronectomy of the second premolar was performed at the crestal level of the alveolar ridge using a surgical motor and specialized burs. Subsequently, an osteotomy was conducted to create a standardized 2‐walled box‐shaped bone defect measuring 8 × 6 × 3 mm (mesio‐distal width × apico‐coronal height × oro‐facial depth) in the alveolar ridge [14]. A dental implant (SPI Element 4.0 × 11 mm MC, Thommen Medical, Grenchen, Switzerland) was then centrally placed in the defect per the manufacturer's recommendations, with the implant contour positioned 1 mm within the bony housing and the implant platform aligned with the lingual bone crest.

### 2.3 GBR, Membrane Stabilization, and Primary Wound Closure

The bone defect was augmented following the principles of GBR, filling and overcontouring the horizontal defect by 2 mm with particulate demineralized bovine bone material (BioOss, Geistlich Pharma AG, Wolhusen, Switzerland) soaked in a radiocontrast agent (Gastrolux, Sanochemia Pharmazeutika, Neufeld an der Leitha, Austria) and covered with a native collagen membrane (Biogide, Geistlich Pharma AG, Wolhusen, Switzerland). For the MDT groups, the collagen membrane was tucked into the previously created buccal periosteal pouch. If the site was randomized to PS, absorbable vertical mattress sutures (Novosyn 5‐0, B. Braun, Melsungen, Germany) were placed mesially and distally to the augmented site. These sutures connected the periosteum apically to the horizontal releasing incision and the lingual mucoperiosteal flap, securing the collagen membrane in place [16].

A single interrupted suture (Optilene 5‐0, B. Braun, Melsungen, Germany) was placed in the midcrestal portion of the flap to facilitate the use of a spring dynamometer (Präzisionsdynamometer, 3B Scientific GmbH, Hamburg, Germany). The flap was tested for primary wound closure under a standardized tension of 0.1 N. If the initial flap advancement was insufficient, further release was achieved by continuing stretching (MPRI) or detachment (MDT) until the flap tension was equal to or less than 0.1 N, allowing for primary wound closure [18]. The buccal flap advancement (FA) at 0.1 N was then measured relative to the gingival margin at the mesial and distal adjacent teeth using a periodontal probe, and the mean of these two measurements was calculated.

A baseline Cone Beam Computed Tomography (CBCT) was taken before primary wound closure (8 × 8 cm, 80 kV, 6 m As, PaX‐Reve3D, Vatech, Hwaseong‐si, South Korea). A board‐certified periodontist, who was not aware of the study scope (E.A.C.) then performed primary wound closure using one horizontal mattress suture at the crest and seven single interrupted sutures along the flap (Optilene 5‐0, B. Braun, Melsungen, Germany). Then, a postoperative CBCT of the hemimandible was taken using the same baseline exposure parameters. Afterward, the sutures and all grafting materials were removed, and the defect was thoroughly rinsed with saline solution. The site was then prepared for a second GBR procedure, with the use of periosteal sutures for membrane stabilization randomized between the first and second procedures.

### 2.4 Digital Measurements

**FIGURE 2:**
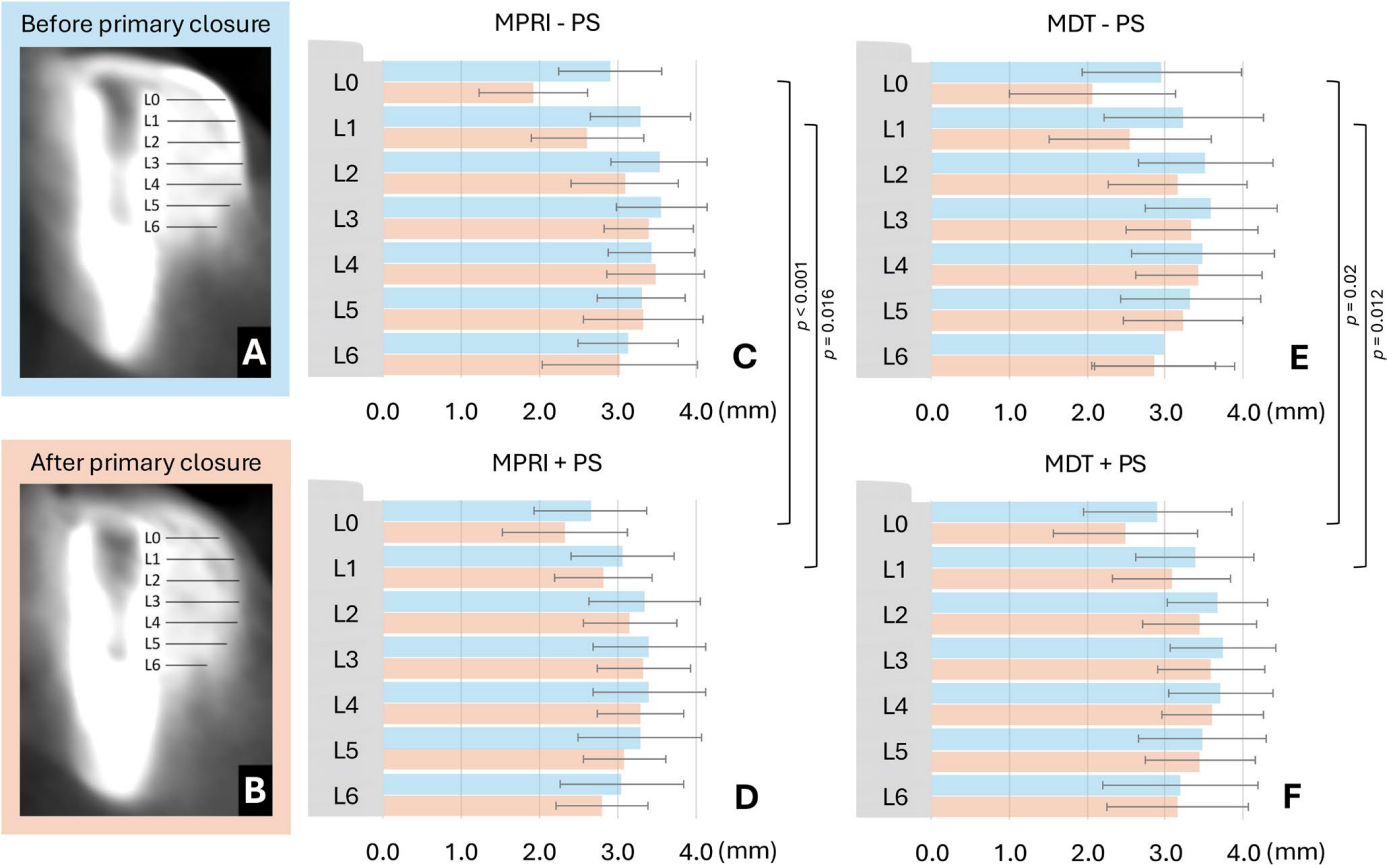
Representative cross‐sectional cone‐beam computed tomography reconstruction from the MDT+PS group before (A) and after primary wound closure (B), including the measurements at different apico‐crestal levels (L0‐L6). Bar plots (± SD) displaying the mean GMT at different apico‐crestal levels (L0‐L6) for MPRI ± PS (C/D) and MDT ± PS (E/F) before and after primary closure. Abbreviations: GMT, graft material thickness; MDT, mucosal detachment technique; MPRI, modified periosteal releasing incision; PS, periosteal suturing.

The displacement of the graft material was assessed in the CBCT datasets, which were analyzed using specialized software (byzz nxt, version 10.2.121, orangedental, Biberach, Germany). Cross‐sectional images, aligned perpendicular to the mandibular curve and the implant axis, were used for evaluation. To standardize measurements across all experimental sites, a transparent acetate foil displaying the implant outline and measurement levels was placed on the monitor [19]. The horizontal graft material thickness before (GMT_0) and after primary wound closure (GMT_1), involving both membrane and bone substitute, were measured perpendicular to the implant surface in 1 mm increments, starting at the implant platform at the crest level and extending to 6 mm below the crest (L0–L6, Figure 2). The absolute and relative differences in GMT before and after primary wound closure were then calculated (ΔGMT mm/%). All CBCT measurements were performed by a single calibrated investigator (C.R.), with half of the dataset re‐assessed after 1 month to calculate intra‐examiner agreement.

### 2.5 Sample Size Calculation and Statistical Analysis

The calculation of the sample size was based on the effect size considered from a previous study employing similar test and control measures [14]. Group sample sizes of 17 were deemed necessary to achieve an 80% power to reject the null hypothesis of equal means when the population mean difference was set at 0.6 with a standard deviation for both groups of 0.5 and with a significance level (alpha) set at 0.05 using ANOVA test.

Because of the non‐parametric nature of the data, the Kruskal–Wallis test was employed to compare differences across multiple groups. For comparisons between the two groups, the Wilcoxon–Mann–Whitney *U* test was used to determine differences. Then Spearman's rank correlation was employed to assess correlations between variables. Additionally, intra‐examiner reliability was evaluated using the intra‐class correlation coefficient [20] after a 1‐month interval, ensuring the consistency and reliability of the measurements.

## 3 Results

In total, 68 GBR procedures (*n* = 17 MDT + PS, *n* = 17 MDT‐PS, *n* = 17 MPRI+PS, *n* = 17 MPRI‐PS) were equally randomized to standardized 2‐walled horizontal bone defects in 34 pig hemimandibles. The intra‐class correlation coefficient for the linear metric digital measurements accounted for 0.94, indicating a high level of consistency between the examiner's measurements at different time points.

### 3.1 Flap Advancement Technique

Both MPRI and MDT resulted in similar flap advancements of 8.3 ± 1.1 mm and 8.3 ± 1.5 mm, respectively. The overall mean relative ΔGMT for MPRI versus MDT amounted to −24.3% ± 13.7% versus −23.0% ± 14.3% at L0, and −14.7% ± 10.4% versus −15.1% ± 10.9% at L1, respectively. There were no statistically significant differences in ΔGMT between MPRI and MDT in the different levels of assessment (*p* ≥ 0.085). Descriptive statistics for GMT and the results of the inter‐group comparison by means of the Wilcoxon test are presented in Tables 1 and 2.

**TABLE 1:** Descriptive statistics (means ± standard deviation) of the flap advancement techniques (MPRI, MDT) and modalities of membrane stabilization (PS, no PS) at different apico‐crestal levels (L0‐L6).

Group	Level	GMT_0 (mm)	GMT_1 (mm)	ΔGMT (mm)	ΔGMT (%)
MPRI	L0	2.8 ± 0.7	2.1 ± 0.7	−0.7 ± 0.3	−24.3 ± 13.7
(MPRI‐PS, MPRI+PS)	L1	3.2 ± 0.6	2.7 ± 0.7	−0.5 ± 0.3	−14.7 ± 10.4
	L2	3.4 ± 0.7	3.1 ± 0.6	−0.3 ± 0.3	−8.6 ± 9.6
	L3	3.5 ± 0.7	3.4 ± 0.6	−0.1 ± 0.3	−2.5 ± 10
	L4	3.4 ± 0.6	3.4 ± 0.6	0 ± 0.4	−0.2 ± 10.2
	L5	3.3 ± 0.7	3.2 ± 0.6	−0.1 ± 0.5	−1.4 ± 14.6
	L6	3.1 ± 0.7	2.9 ± 0.8	−0.2 ± 0.7	−4 ± 16.3
MDT	L0	2.6 ± 1	2.3 ± 1	−0.6 ± 0.4	−23 ± 14.3
(MDT‐PS, MDT+PS)	L1	3.1 ± 0.9	2.8 ± 0.9	−0.5 ± 0.4	−15.1 ± 10.9
	L2	3.5 ± 0.8	3.3 ± 0.8	−0.3 ± 0.4	−8.3 ± 10.3
	L3	3.6 ± 0.8	3.5 ± 0.8	−0.2 ± 0.4	−5.5 ± 9.2
	L4	3.5 ± 0.8	3.5 ± 0.7	−0.1 ± 0.3	−1.3 ± 9.5
	L5	3.3 ± 0.9	3.3 ± 0.7	−0.1 ± 0.3	−0.8 ± 8.4
	L6	3 ± 0.9	3 ± 0.9	−0.1 ± 0.3	−1.6 ± 9.9
No PS	L0	2.9 ± 0.8	2 ± 0.6	−0.9 ± 0.5	−33.2 ± 16.9
(MPRI‐PS, MDT‐PS)	L1	3.3 ± 0.8	2.6 ± 0.6	−0.7 ± 0.5	−21.3 ± 14.3
	L2	3.5 ± 0.7	3.1 ± 0.5	−0.4 ± 0.4	−11.5 ± 11
	L3	3.6 ± 0.7	3.4 ± 0.5	−0.2 ± 0.4	−5.4 ± 10.2
	L4	3.5 ± 0.7	3.5 ± 0.5	0 ± 0.4	0.8 ± 10.5
	L5	3.3 ± 0.7	3.3 ± 0.5	0 ± 0.4	−0.5 ± 11.2
	L6	3.1 ± 0.8	2.9 ± 0.6	−0.1 ± 0.5	−3 ± 12.4
PS	L0	2.8 ± 0.8	2.4 ± 0.9	−0.4 ± 0.3	−14 ± 11.1
(MPRI+PS, MDT+PS)	L1	3.2 ± 0.7	3 ± 0.7	−0.3 ± 0.2	−8.5 ± 7
	L2	3.5 ± 0.7	3.3 ± 0.7	−0.2 ± 0.3	−5.4 ± 8.9
	L3	3.6 ± 0.7	3.5 ± 0.6	−0.1 ± 0.3	−2.5 ± 9
	L4	3.6 ± 0.7	3.5 ± 0.6	−0.1 ± 0.3	−2.3 ± 9.1
	L5	3.4 ± 0.8	3.3 ± 0.6	−0.1 ± 0.4	−1.6 ± 11.8
	L6	3.1 ± 0.9	3 ± 0.8	−0.1 ± 0.4	−2.6 ± 13.8
MPRI‐PS	L0	2.9 ± 0.7	1.9 ± 0.7	−1 ± 0.4	−35.1 ± 14.9
	L1	3.3 ± 0.6	2.6 ± 0.7	−0.7 ± 0.4	−21.4 ± 14
	L2	3.5 ± 0.6	3.1 ± 0.7	−0.4 ± 0.4	−12.6 ± 10.8
	L3	3.6 ± 0.6	3.4 ± 0.6	−0.2 ± 0.4	−3.9 ± 10.2
	L4	3.4 ± 0.6	3.5 ± 0.6	0.1 ± 0.4	1.8 ± 9.7
	L5	3.3 ± 0.6	3.3 ± 0.8	0 ± 0.5	0.6 ± 13.5
	L6	3.1 ± 0.6	3 ± 1	−0.1 ± 0.8	−2.9 ± 14.3
MPRI+PS	L0	2.7 ± 0.7	2.3 ± 0.8	−0.3 ± 0.3	−13.5 ± 12.4
	L1	3.1 ± 0.7	2.8 ± 0.6	−0.3 ± 0.2	−8.1 ± 6.8
	L2	3.3 ± 0.7	3.2 ± 0.6	−0.2 ± 0.2	−4.5 ± 8.4
	L3	3.4 ± 0.7	3.3 ± 0.6	−0.1 ± 0.3	−1 ± 9.8
	L4	3.4 ± 0.7	3.3 ± 0.6	−0.1 ± 0.4	−2.1 ± 10.7
	L5	3.3 ± 0.8	3.1 ± 0.5	−0.2 ± 0.6	−3.3 ± 15.8
	L6	3.1 ± 0.8	2.8 ± 0.6	−0.3 ± 0.6	−5 ± 18.2
MDT‐PS	L0	3 ± 1	2.1 ± 1.1	−0.9 ± 0.6	−31.4 ± 18.8
	L1	3.2 ± 1	2.6 ± 1	−0.7 ± 0.5	−21.2 ± 14.7
	L2	3.5 ± 0.9	3.2 ± 0.9	−0.4 ± 0.4	−10.4 ± 11.1
	L3	3.6 ± 0.9	3.3 ± 0.8	−0.3 ± 0.4	−7 ± 10.2
	L4	3.5 ± 0.9	3.4 ± 0.8	0 ± 0.4	−0.1 ± 11.3
	L5	3.3 ± 0.9	3.2 ± 0.8	−0.1 ± 0.3	−1.6 ± 9
	L6	3 ± 0.9	2.9 ± 0.8	−0.1 ± 0.3	−3.1 ± 10.5
MDT+PS	L0	2.9 ± 0.9	2.5 ± 0.9	−0.4 ± 0.3	−14.6 ± 9.9
	L1	3.4 ± 0.8	3.1 ± 0.8	−0.3 ± 0.3	−9 ± 7.1
	L2	3.7 ± 0.6	3.4 ± 0.7	−0.2 ± 0.4	−6.2 ± 9.4
	L3	3.7 ± 0.7	3.6 ± 0.7	−0.2 ± 0.3	−4 ± 8.2
	L4	3.7 ± 0.7	3.6 ± 0.6	−0.1 ± 0.3	−2.5 ± 7.6
	L5	3.5 ± 0.8	3.5 ± 0.7	0 ± 0.2	0.1 ± 7.8
	L6	3.2 ± 1	3.2 ± 0.9	0 ± 0.3	−0.1 ± 9.3

Abbreviations: GMT, Graft material thickness; MDT, Mucosal detachment technique; MPRI, Modified periosteal releasing incision; PS, Periosteal suturing.

**TABLE 2:** Results of the inter‐group Wilcoxon test comparing the flap advancement techniques (MPRI, MDT) and modalities of membrane stabilization (PS, no PS) at different apico‐crestal levels (L0‐L6).

Level	MPRI versus MDT	No PS versus PS	MPRI‐PS versus MDT‐PS	MPRI+PS versus MDT+PS	MPRI‐PS versus MPRI+PS	MDT‐PS versus MDT+PS
L0	0.67	< 0.001*	0.267	0.540	< 0.001*	0.020*
L1	0.93	< 0.001*	0.913	0.965	0.016*	0.012*
L2	0.085	0.041*	0.846	0.913	0.543	0.342
L3	0.24	0.41	1.000	0.899	0.729	0.755
L4	0.23	0.12	0.549	0.661	0.499	0.562
L5	0.6	0.81	1.000	0.843	1.000	1.000
L6	0.73	0.68	1.000	1.000	0.997	1.000

Abbreviations: MDT, Mucosal detachment technique; MPRI, modified periosteal releasing incision; PS, periosteal suturing. *p‐value < 0.05.

### 3.2 Membrane Stabilization

The overall mean relative ΔGMT for PS versus no PS in L0 to L2 amounted to −14.0% ± 11.1% versus −33.2% ± 16.9% (*p* < 0.001) at L0, −8.5% ± 7.0% versus −21.3% ± 14.3% (*p* < 0.001) at L1, and −5.4% ± 8.9% versus −11.5% ± 11.0% (*p* = 0.041) at L2, respectively. There were no statistically significant differences in ΔGMT between PS and no PS in L3–L6 (*p* ≥ 0.12). Descriptive statistics for GMT and the results of the Wilcoxon test are presented in Tables 1 and 2.

### 3.3 Interaction Term Analysis

When comparing the two flap advancement techniques based on membrane stabilization, no significant differences were observed between the MPRI‐PS and MDT‐PS subgroups (*p* ≥ 0.267) or between MPRI+PS and MDT+PS (*p* ≥ 0.54) in any of the levels. In contrast, when evaluating the two methods of membrane stabilization within each flap advancement technique, significantly less graft material displacement was observed for PS than no PS in both the MPRI‐PS versus MPRI+PS (*p* < 0.016) and MDT‐PS versus MDT+PS (*p* < 0.02) comparisons in L0 and L1, but not in L2‐L6 (*p* ≥ 0.342).

### 3.4 Phenotypic Characteristics

The mean KMW amounted to 6.8 ± 0.9 mm, and the mean FT3, FT6, and FT9 were 1.6 ± 0.5 mm, 1.0 ± 0.6 mm, and 0.8 ± 0.3 mm, respectively. No statistically significant differences regarding KMW, FT3, FT6, and FT9 were found on ΔGMT (*p* ≥ 0.238) or the amount of flap advancement for MPRI and MDT (*p* ≥ 0.264). Descriptive statistics of the local soft tissue phenotypical characteristics are presented in Table 3.

**TABLE 3:** Descriptive statistics (means ± standard deviation) of the local phenotypical soft tissue for the two flap advancement techniques. MPRI: Modified periosteal releasing incision. MDT: Mucosal detachment technique. KMW: Keratinized mucosa width. FT3/6/9: Flap thickness 3/6/9 mm apically from the incision line. FA: Flap advancement after periosteal release.

	KMW (mm)			FT3 (mm)			FT6 (mm)			FT9 (mm)			FA (mm)		
MPRI	6.9	±	0.8	1.7	±	0.6	1.1	±	0.6	0.9	±	0.2	8.3	±	1.1
MDT	6.7	±	1.1	1.4	±	0.4	1.0	±	0.5	0.8	±	0.4	8.3	±	1.5

## 4 Discussion

This ex vivo study demonstrated a significant graft material displacement at the level of the implant crest during primary wound closure in GBR procedures for two different flap advancement techniques. Analysis of GMT changes revealed that additional membrane stabilization using PS resulted in significantly less graft material displacement as compared with no PS. Finally, both flap advancement techniques were equally effective and not influenced by the soft tissue phenotypical characteristics. Therefore, H01 and H03 were rejected, while H02 was not.

Flap design and advancement are fundamental prerequisites for the treatment success of regenerative tissue volume augmentation procedures in dental implant therapy. The flap advancement techniques investigated in the present study are distinguished by the presence of a periosteal pouch, which might be utilized for graft material deposition. Both techniques allowed for horizontal bone augmentation with a difference in GMT before and after primary wound closure of −35.1% ± 14.9%, and −31.4% ± 18.8% at implant crest for the MPRI‐PS and MDT‐PS technique, respectively. Interestingly, this difference was not significant, indicating that the flap advancement technique does not affect the displacement of the graft material during primary wound closure. These results are slightly lower than the −42.8% ± 17.9% difference reported in another study on GBR procedures utilizing particulate graft materials and collagen membranes, where the use of a block‐type graft material or two pins for membrane stabilization reduced dimensional changes to −20.2% ± 18.9% and −22.9% ± 21.2%, respectively [14]. Despite their effectiveness, the use of pins for graft material stabilization has disadvantages, which might be mitigated by using periosteal mattress sutures as an alternative [16].

In the present study, the use of PS resulted in a difference in GMT before and after primary wound closure of −13.5% ± 12.4%, and −14.6% ± 9.9% for the MPRI+PS, and MDT+PS techniques, respectively. These values are slightly lower than the above‐mentioned values reported for membrane stabilization using two pins in the apical region, demonstrating the effectiveness of PS in graft material stabilization. A recent pilot study investigated the increase in bone thickness of staged GBR procedures using periosteal mattress sutures using preoperative and 6‐month‐follow‐up CBCT datasets in six patients. The authors reported a significant increase in bone thickness of 3.4 ± 1.3 mm and uneventful wound healing in all cases [21]. However, the study did not investigate the efficacy of PS in graft material stabilization during the intraoperative wound closure phase. Even though the biodegradation intervals for the different materials of absorbable sutures are provided by the manufacturers, operators should also consider the potential tearing of the knots or stretching of the periosteum over time. These factors potentially limit the predictability of PS on membrane stabilization during GBR as compared with the use of pins.

Both the MDT and MPRI techniques resulted in comparable and adequate flap advancements of 8.3 ± 1.5 mm and 8.3 ± 1.1 mm, respectively. In a similar setting, flap advancements of 8.77 ± 0.25 mm and 3.78 ± 0.37 mm were reported for the MDT and PRI without lateral stretching, respectively (L. [17]). These values are close to the range of the 5.5 ± 1.5 mm flap advancement observed in a prospective cohort study investigating the effect of vertical and PRI techniques in 30 patients undergoing dental implant surgeries [22]. Throughout the literature, the assessment of flap advancement was conducted using forces ranging from no tension up to 1 N [18, 22, 23, 24]. However, from a clinical point of view, closing forces higher than 0.1 N were found to significantly increase the amount of postoperative wound dehiscence defects [18]. Therefore, this value was used as a threshold for the measurement of flap advancement in the present study. Applying forces other than 0.1 N might lead to different flap advancement measures. Nevertheless, both MDT and MPRI appear to facilitate tension‐free primary wound closure, which is essential for avoiding post‐surgical complications. Adequate flap advancement helps to prevent the occurrence of wound dehiscences, reducing the risk of infections, partial graft loss, and suboptimal outcomes in regenerative procedures [5].

Notably, the phenotypic characteristics of soft tissues, including KMW and FT, did not influence the extent of flap advancement or graft material displacement in the cadaveric model used in the present study. Nevertheless, preoperative assessment of KMW and mucosal thickness, utilizing either invasive or non‐invasive techniques, is essential for treatment planning to achieve aesthetic and long‐term successful outcomes [25]. Additionally, the soft tissue phenotype may influence the risk of developing postoperative wound dehiscence, as indicated by a clinical trial that observed a tendency for increased dehiscence in thin soft tissue phenotypes when flap tensions exceeded 0.1 N [18].

The reliability and reproducibility of the pig mandible in an ex vivo study design for the assessment of graft material displacement during wound closure have been proven in previous investigations focusing on different GBR materials [13, 14, 15]. Nevertheless, care should be taken when interpreting the present study results because of the following limitations: first, only two flap advancement techniques within one flap design, with and without PS, were investigated. Other surgical techniques or methods of graft material stabilization might result in different amounts of GMT and FA. Second, although meticulous care was taken to ensure that primary wound closures during this investigation did not exceed the threshold for flap tensions of 0.1 N, potential confounding factors on graft material displacement might still exist within the range of flap tensions below 0.1 N. Third, while the defect dimensions were standardized, the quantity or the condensation of the graft material was not. This may have influenced the results, as the impact of graft material condensation on its displacement remains uncertain. Fourth, the ex vivo design does not account for confounding factors affecting graft material displacements, such as postoperative swelling and hematoma formation, mucosal movements during mastication or facial expressions, or physical touch by the patient. Future investigations might explore the impact of the number of pins or the use of fibrin sealant on graft material displacement during primary wound closure. Additionally, the durability of the stabilizing effect of PS remains an area requiring further research.

## 5 Conclusions

Within the limitations of the present study, it can be concluded that:

1. MPRI and MDT result in similar amounts of graft material displacement during primary wound closure.
2. PS is effective in reducing graft material displacement during primary wound closure.
3. Both MPRI and MDT are effective techniques for flap advancement.
4. Graft material displacement and flap advancement are not influenced by KMW or FT.

## Author Contributions

**C.R.** and **F.S.:** conceived the idea and designed the study. **C.R., E.A.C.,** and **F.S.:** acquired and analyzed the data. **C.R.:** led the writing. **C.R., E.A.C., E.C.Q., V.C., A.R.,** and **F.S.:** contributed to data interpretation and critically revised the manuscript. All authors gave final approval and agreed to be accountable for all aspects of the scientific work.

## Ethics Statement

The authors have nothing to report.

## Conflicts of Interest

The authors declare no conflicts of interest.

## Data Availability

The data that support the findings of this study are available from the corresponding author upon reasonable request.
